# An Integrated Workflow for Long-Term Fiber Photometry Analysis

**DOI:** 10.1523/ENEURO.0128-26.2026

**Published:** 2026-10-06

**Authors:** Farina Pourmir, Jordan N. Cook, Samantha O. Sweck, Jeff R. Jones

**Affiliations:** ^1^Department of Biology, Texas A&M University, College Station, Texas 77843; ^2^Institute for Neuroscience, Texas A&M University, College Station, Texas 77843; ^3^Center for Biological Clocks Research, Texas A&M University, College Station, Texas 77843

**Keywords:** calcium imaging, circadian rhythms, fiber photometry, long-term recording, open-source software, signal analysis

## Abstract

Long-term fiber photometry enables measurement of neural dynamics across hours to days, but these recordings create analytical and reproducibility challenges that are not well addressed by tools developed for short, stimulus-locked experiments. Here we present a software environment for long-term photometry analysis organized around a structured guided workflow for configuring, reviewing, executing, and inspecting analyses. The workflow makes consequential analysis choices visible by allowing correction strategies and event-detection settings to be previewed and configured for individual regions of interest before analysis. It supports both intermittent and continuous recordings and provides complementary views of corrected Δ*F*/*F*, detected-event summaries, and slow-signal structure across session-level and multiday timescales. We illustrate how correction choice can substantially alter the resulting Δ*F*/*F* under challenging signal-reference conditions. We also demonstrate long-term analysis of recordings obtained with calcium, acetylcholine, and dopamine sensors in male mice. Together, these capabilities provide a practical framework for reproducible analysis of long-term fiber photometry recordings.

## Significance Statement

Most fiber photometry analysis tools were developed for short, event-driven experiments rather than for recordings collected over hours, days, or even weeks. Long-term datasets can contain hundreds or thousands of recording intervals, making exhaustive trace-by-trace inspection impractical and increasing the importance of consistent approaches to signal correction, event detection, and temporal organization. We present a guided software workflow that makes these analysis choices visible and reviewable while organizing long-duration recordings into interpretable session-level and multiday outputs.

## Introduction

Genetically encoded fluorescent indicators have expanded the temporal reach of systems neuroscience by making it possible to monitor neural signals across hours, days, and, in some cases, weeks (Tang et al., 2025). Many fundamental physiological and behavioral processes unfold over these longer timescales, shaped by internal state, environmental context, and recurring biological rhythms rather than by brief stimulus-evoked events (Hrvatin et al., 2020; Rinker et al., 2023; Yaguchi et al., 2023; Chen et al., 2024; Vandendoren et al., 2024; Vas et al., 2024). Circadian systems make the importance of these longer timescales especially clear, because their underlying neural and molecular dynamics unfold across the full 24 h cycle (Jones et al., 2018, 2021; Sayar-Atasoy et al., 2023; Douglass et al., 2024; Gomez et al., 2025; Yurgel et al., 2025). More broadly, the same need for extended observation applies to circuits whose activity changes slowly or recurrently over time. Long-term optical recording therefore offers direct access to aspects of neural function that were previously difficult to study.

Analytical assumptions that work well in short, stimulus-locked recordings often break down in long-term photometry datasets. Over these longer timescales, fluorescence reflects a mixture of biological signal, drift, photobleaching, motion-related artifacts, and other biological or technical influences that complicate interpretation (Simpson et al., 2023). As a result, similar datasets can yield divergent conclusions when they are analyzed under different assumptions or through poorly standardized workflows. Long-term photometry therefore poses a problem of interpretation and reproducibility at the level of the analytical workflow itself.

In our prior work, we used circadian photometry as a case study to argue that long-term optical monitoring requires analytical strategies tailored to extended recordings (Tang et al., 2025). That paper emphasized reproducibility and standardization and introduced tonic and phasic decomposition to distinguish slow and transient components of long-duration fluorescence signals (Enoki et al., 2017). It further argued that common analysis practices often treat meaningful slow structure as baseline drift or otherwise blur the distinct information carried by those signal components. These concepts established an analytical framework for long-term photometry, but implementing that framework across extended datasets poses additional practical challenges.

Long-term recordings also create practical analysis problems that become increasingly important as recordings extend across days. Intermittent experiments may contain hundreds or thousands of individual recording intervals, while continuous recordings require analysis and visualization over equally long uninterrupted timelines. In either case, exhaustive trace-by-trace inspection is impractical, yet signal-correction choices can substantially alter the corrected trace, while event-detection and temporal-organization choices shape the quantitative summaries and long-duration views derived from it. Existing open-source tools support important components of fiber photometry analysis, particularly for shorter experiments, but they do not fully address the combination of extended recording duration, large numbers of recording intervals, and the need to review consequential correction and event-detection choices across the dataset (Bruno et al., 2020; Sherathiya et al., 2021; Conlisk et al., 2023; Keevers and Jean-Richard-Dit-Bressel, 2025).

Here we present a guided software workflow developed specifically for fiber photometry recordings that extend across hours to days. The software organizes data import, definition of recording structure, region of interest (ROI)-specific correction, event detection, analysis, and result review into a defined sequence, with visual previews at stages where analytical choices can materially alter the corrected signal or downstream analysis outputs. We use this framework to examine correction behavior under changing or unreliable signal–reference relationships, to demonstrate analysis of both intermittent and continuous recordings, and to apply the workflow to long-term recordings from calcium, acetylcholine, and dopamine sensors.

## Materials and Methods

### Animals and housing

Male wild-type (*n* = 4) and ChAT-IRES-Cre [*n* = 2; (Rossi et al., 2011)] mice, 3 months old, on a C57BL/6j background were used for the recordings included in this study. Mice were singly housed at constant temperature (∼23°C) and humidity (∼40%) under either a 12:12 h light/dark cycle (LD; lights on defined as zeitgeber time 0) or in constant darkness (DD), with food and water available *ad libitum*. All procedures were approved by the Texas A&M University Institutional Animal Care and Use Committee and performed in accordance with institutional guidelines.

### Stereotaxic surgery

Mice were anesthetized with isoflurane, and buprenorphine-SR (1 mg/kg, s.c.) was administered before surgery. After the skull was leveled, viral vectors were injected unilaterally according to the experimental group. Two wild-type mice received hippocampal injections of AAV9-hSyn-gACh4m (∼1.17 × 10^13^ GC/ml; BrainVTA; Gangal et al., 2025) at −2.00 mm posterior, +1.25 mm lateral, and −1.25 mm ventral relative to bregma, at a volume of 100 nl and a rate of 1 nl/min. Two wild-type mice received nucleus accumbens injections of AAV9-hSyn-DA3m (∼5.40 × 10^12^ GC/ml; BrainVTA; Cook et al., 2026), diluted 1:4 in phosphate-buffered saline, at +1.42 mm anterior, +0.75 mm lateral, and −4.70 mm ventral relative to bregma, at a volume of 200 nl and a rate of 1 nl/min. Two ChAT-IRES-Cre mice received medial septum injections of AAVDJ/8-hSyn-DIO-GCaMP7s (∼8.80 × 10^12^ GC/ml; University of Zurich Viral Vector Facility; Zhang et al., 2023) at +0.85 anterior, 0.00 mm lateral, and −4.35 mm ventral relative to bregma, at a volume of 100 nl and a rate of 1 nl/min. The injection needle was left in place for 10 min before withdrawal. A fiber-optic cannula (2 or 6 mm length, 200 µm core diameter, 0.39 NA; RWD Life Science) was implanted at the corresponding injection site and secured with adhesive cement (Parkell) and opaque black dental cement. The implant was covered with a thin layer of black nail polish. Mice recovered in their home cages for 2 weeks before experiments.

### Fiber photometry

Fiber photometry data were collected using a multichannel fiber photometry system (R821; RWD Life Science). After postsurgical recovery, mice were tethered to the photometry system and allowed at least 2 d to acclimate to the patch cable (200 µm core diameter, 0.37 NA, 3 m length; Doric Lenses) before recordings began. Fluorescence was sampled at 40 frames/s using interleaved 470 nm signal and 415 nm reference excitation channels with a 33% duty cycle (10 min on, 20 min off).

### Recording structure and temporal alignment

The software supports intermittent and continuous recording structures. Intermittent recordings consist of discrete recording sessions acquired at known times across the experiment, whereas continuous recordings consist of a single uninterrupted fluorescence time series. The biological recordings analyzed in this study were acquired intermittently using the 33% duty cycle described above, yielding two 10 min recording sessions per hour across multiday experiments. Individual sessions retained their position within the experimental timeline rather than being concatenated sequentially, with temporal alignment defined in elapsed time, clock time, or relative to a user-defined daily reference time. The synthetic continuous datasets used in this study consisted of uninterrupted fluorescence time series spanning the full simulated interval.

### Software implementation and supported inputs

The software was implemented in Python with a PySide6 graphical user interface and executes analyses locally on the user's computer. The software is currently documented for use on Windows. Once the application is launched, analyses can be configured and executed through the graphical interface without command-line interaction.

The guided workflow supports RWD recordings, Neurophotometrics recordings, and user-mapped CSV files. RWD recordings can be analyzed as either intermittent or continuous recordings, while Neurophotometrics recordings can be analyzed as intermittent recordings. For CSV input, the user identifies the time column and its units and explicitly maps signal and reference columns for each ROI. A single mapped CSV file can be analyzed as a continuous recording, whereas multiple mapped CSV files can be treated as intermittent sessions. Other acquisition formats require export to a CSV representation that can be mapped to the required time, signal, and reference fields.

ROI and channel identities are established according to the input format. For RWD recordings, ROIs are identified from paired fluorescence-channel names. For Neurophotometrics recordings, ROIs are identified from recorded Region columns, with LED states used to distinguish signal and reference samples. For mapped CSV input, ROI identities are defined by the user-specified signal and reference column pairs. Multiple ROIs can be included in a single analysis, and individual ROIs can be included or excluded before execution.

Before analysis, the software validates the selected source, ROI and channel mappings, timestamp structure, sampling cadence, recording structure, and completeness of the analysis configuration. Invalid or ambiguous channel assignments, nonnumeric or nonfinite timestamps, duplicated or decreasing timestamps, and unresolved recording structure inconsistencies prevent execution.

### Guided analysis workflow and analysis provenance

Photometry analyses are configured through the guided workflow described in Figure 1. After selecting the input data, the user identifies the included ROIs and defines the recording structure and temporal alignment before configuring correction and event detection. A correction strategy is confirmed separately for each included ROI, with eligible correction parameters also configurable by ROI. Event-detection settings are defined from a common set of defaults, with optional ROI-specific adjustments. Before proceeding to Run, the user reviews the input data, recording structure, and timing, included ROIs, correction choices, event-detection settings, and output location.

Correction previews are used to compare candidate strategies on a selected recording segment before confirmation. The fitted model produced during preview is not reused in the final analysis; after a strategy is confirmed, correction is re-estimated from the production data. For intermittent recordings, correction models are estimated separately for each recording session; for continuous recordings, they are estimated within successive correction segments. Event-detection settings can likewise be previewed on a selected segment, but events identified during preview are not retained as final analysis results.

Before execution, Setup Check verifies that the required input data, recording structure, ROI selections, correction and event-detection settings, temporal alignment, and output location are complete and internally consistent. The Run action performs a final consistency check. Changes to the input source or relevant analysis settings after validation require Setup Check to be repeated before execution.

Analyses completed with the guided workflow preserve the recording identity and structure, including ROIs, per-ROI correction strategies and parameters, per-ROI event-detection settings, temporal configuration, software build identity, and analysis outputs. When a completed analysis is reopened, the application loads the saved results and analysis settings associated with that run rather than applying current interface defaults. Reopening the analysis does not recompute the original correction or event detection. Figures and visualizations are saved as PNG files, while quantitative outputs are saved in CSV format for use in downstream statistical and programming workflows.

### Signal correction and ΔF/F calculation

Signal correction is performed independently for each included ROI using the correction strategy selected during the guided workflow. Three strategies, global linear regression, Robust Global Event-Reject Fit, and adaptive event-gated regression, use the simultaneously recorded reference channel to estimate a correction reference. Signal-Only F0 instead estimates a baseline directly from the signal channel and does not use the reference channel.

For the three reference-based strategies, let $S_{j}(t)$ denote the raw signal trace for recording session or correction segment j, and let ${\hat{R}}_{j}(t)$ denote the fitted correction-reference trace for that interval. The corrected signal is calculated as follows:

$$\Delta F_{j}(t)=S_{j}(t)-{\hat{R}}_{j}(t)\;.$$

Normalization is performed separately from estimation of the correction reference. A separate recording-wide robust signal–reference model is fit once for each ROI to obtain ${\hat{R}}_{\mathrm{norm}}(t)$. For each recording session or correction segment j, $F_{0j}$ is defined as the median of the finite values of ${\hat{R}}_{\mathrm{norm}}(t)$ within that interval. The final normalized signal is then as follows:

$${\left(\frac{\Delta F}{F}\right)}_{j}(t)=100\frac{\Delta F_{j}(t)}{F_{0j}}.$$

Thus, the selected correction strategy determines the numerator, whereas a common recording-wide reference model determines the interval-specific scalar denominator. Samples excluded or gated during estimation of the correction reference are retained in the final trace after the fitted reference has been reconstructed.

For global linear regression, both signal and reference traces are low-pass filtered using a third-order, 1 Hz Butterworth filter applied with zero-phase filtering to yield $S_{\mathrm{filt},j}(t)$ and $R_{\mathrm{filt},j}(t)$. Within each recording session or correction segment, ordinary least-squares regression is then used to estimate the linear relationship

$$S_{\mathrm{filt},j}(t)=aR_{\mathrm{filt},j}(t)+b,$$

where a and b are the fitted slope and intercept, from all finite filtered signal–reference pairs. The resulting coefficients are applied to the raw reference trace, $R_{\mathrm{raw},j}(t)$, to obtain the fitted correction reference:

$${\hat{R}}_{j}(t)=aR_{\mathrm{raw},j}(t)+b.$$

The raw signal and fitted correction reference are then used to calculate Δ*F* and the normalized Δ*F*/*F* trace as described above.

For Robust Global Event-Reject Fit, a robust linear model is estimated independently within each recording session or correction segment from the raw signal and reference values using Huber regression. Residuals are calculated as the difference between the raw signal and fitted reference, centered on the residual median, and scaled using 1.4826×MAD. Samples whose residual robust-*z* values exceed the selected threshold are excluded from subsequent fitting passes, and the model is iteratively refit until the retained-sample mask converges, the maximum number of iterations is reached, or further rejection would reduce the retained fraction below the specified minimum. The default settings are three fitting iterations, a residual robust-*z* threshold of 3.5, and a minimum retained fraction of 0.50. If the robust model cannot be estimated, correction falls back to global linear regression. The final robust coefficients are applied to the complete raw reference trace for that session or correction segment, including time points excluded from fitting, to obtain the fitted correction reference.

For adaptive event-gated regression, an initial robust signal-reference model is estimated within each recording session or correction segment and used to identify trusted samples from the residual signal. Residual excursions are identified using median/MAD-based robust-*z* values, with a default threshold of 3.5. Time-varying regression coefficients are then estimated from trusted samples within the same session or correction segment using centered moving-window sums of the signal, reference, squared reference, and signal-reference product. The default moving regression window is 60 s. Local coefficient estimates require sufficient trusted support and nondegenerate reference variance. Missing coefficient estimates are interpolated from neighboring valid estimates, and local estimates are blended toward the initial robust coefficients according to the available trusted support. Across gated spans, coefficients are held using the nearest trusted anchor. At least 50% of the finite signal–reference pairs within the recording session or correction segment must remain trusted. If this criterion cannot be satisfied, correction falls back first to Robust Global Event-Reject Fit and then, if necessary, to global linear regression. The resulting time-varying correction reference is as follows:

$${\hat{R}}_{j}(t)=a(t)R_{\mathrm{raw},j}(t)+b(t),$$

and is evaluated across the complete raw reference trace for that session or correction segment.

For Signal-Only F0, correction is calculated from the raw signal without reference-channel information. A slowly varying signal-derived baseline, B(t), is estimated independently within each intermittent recording session or continuous correction segment. The estimator begins with a rolling 10th-percentile lower envelope calculated over a window equal to 20% of the duration of that session or correction segment, subject to minimum and maximum window-size limits. The resulting envelope is smoothed using a finite-aware moving average with a default window equal to 10% of the session or correction-segment duration. Low-signal portions of the recording are then identified from the smoothed signal using robust percentile-based state classification. Local lower-quantile baseline anchors are generated within these low-signal regions at approximately quarter-window intervals. When at least three valid anchors are available, B(t) is obtained by interpolation between anchors, with the nearest valid anchor held beyond the outermost anchors. If low-signal support or anchor coverage is insufficient, the algorithm falls back to the smoothed rolling-envelope estimate. During prolonged elated-signal states, an additional contextual cap limits the resulting baseline estimate relative to the robust 5th-to-95th-percentile signal range. The corrected and normalized signal is calculated as follows:

ΔF(t)=S(t)−B(t),

and

$$\frac{\Delta F}{F}(t)=100\frac{S(t)-B(t)}{B(t)}.$$

Signal-Only parameters are fixed in the guided workflow rather than exposed as per-ROI correction settings.

### Event detection

Event detection is performed independently for each included ROI using either Δ*F*, defined by the signal minus the fitted correction reference or signal-derived baseline for the selected correction strategy, or the normalized Δ*F*/*F* signal described above. Δ*F*/*F* is used by default. For intermittent recordings, events are detected separately within each recording session. For continuous recordings, the corrected recording is reconstructed as a single contiguous trace for each ROI and event detection is performed across the full recording. Events can be detected as positive excursions, negative excursions, or both. Positive events are identified directly from the detection trace, whereas negative events are identified after sign inversion. When both polarities are selected, positive and negative searches are performed separately, and the resulting event indices are combined and ordered by sample time. Detection thresholds are calculated from the finite samples of the detection trace using one of four methods: mean-based, median-based, percentile, or absolute thresholding. Each method defines an upper threshold for positive-event detection and a lower threshold for negative-event detection.

For mean-based thresholding, the upper and lower detection thresholds are calculated from the trace mean and population standard deviation as follows:

$$T_{\mathrm{upper}}=\;\mu +k\sigma ,$$

and

$$T_{\mathrm{lower}}=\;\mu -k\sigma ,$$

where $T_{\mathrm{upper}}$ and $T_{\mathrm{lower}}$ are the upper and lower detection thresholds, μ and σ are the mean and population standard deviation of the finite detection-trace values, and k is the selected threshold multiplier.

For median-based thresholding, the same structure is used with the median and a robust MAD-based estimate of variability:

$$T_{\mathrm{upper}}=\;m+k(1.4826\times \mathrm{MAD}),$$

and

$$T_{\mathrm{lower}}=\;m-k(1.4826\times \mathrm{MAD}),$$

where m is the median of the finite detection-trace values and MAD is their median absolute deviation from m.

For percentile thresholding, the upper and lower detection thresholds are defined directly from a user-selected percentile p of the finite detection-trace values. The upper threshold is the *p*th percentile and the lower threshold is the complementary (100−p)th percentile. For example, p=95 uses the 95th percentile for positive excursions and the 5th percentile for negative excursions.

For absolute thresholding, the selected absolute threshold is applied symmetrically as positive and negative bounds. Only the upper threshold is used for positive-event detection, only the lower threshold for negative-event detection, and both are used when both polarities are selected.

Peaks are identified using scipy.signal.find_peaks. Minimum distance and minimum width are specified in seconds and converted to sample counts using the sampling rate. Minimum prominence is defined as follows:

$$P_{\min}=k_{p}(1.4826\times \mathrm{MAD}),$$

where $k_{p}$ is the selected prominence multiplier. Width is evaluated at half-prominence. The default guided threshold is mean-based with *k* = 2.5; the default minimum distance is 1 s, prominence multiplier is 2.0, and minimum width is 0.3 s. An optional event-specific prefilter can be applied before threshold calculation and peak detection; the default is no prefilter. When enabled, a third-order, 1 Hz Butterworth low-pass filter is applied with zero-phase filtering and does not alter the stored corrected trace. Nonfinite samples are excluded from threshold calculations and are not interpolated. Detection is performed separately within contiguous finite portions of the trace, so events cannot span nonfinite gaps.

### Event-derived summaries

For intermittent recordings, event count is calculated separately for each recording session, and event rate is calculated as the number of detected events per minute of recording. For continuous recordings, events are detected across the full corrected recording as described above and then assigned to nonoverlapping analysis windows; event rate is calculated separately for each window using its actual duration.

Integrated fluorescence is defined as the area of the configured event signal, either corrected Δ*F* or normalized Δ*F*/*F* as defined above, relative to a selected integration baseline. The calculation spans the full trace for each intermittent recording session or continuous analysis window, excluding nonfinite samples. If the optional event prefilter is enabled, the filtered event signal is integrated; otherwise, the unfiltered corrected event signal is used. Integrated fluorescence is not restricted to samples classified as detected events. Integrated fluorescence is quantified as area under the curve (AUC). If x(t) is the event signal and b is the integration baseline, the positive-polarity calculation is as follows:

AUC=∫max[x(t)−b,0]dt,

the negative-polarity calculation is as follows:

AUC=∫min[x(t)−b,0]dt,

and the both-polarity calculation is as follows:

AUC=∫[x(t)−b]dt.

Negative-polarity AUC therefore remains negative, whereas the both-polarity setting gives signed net area in which positive and negative excursions can offset one another.

The integration baseline is either zero or the median of the finite event signal. For intermittent recordings, a median baseline is calculated separately for each recording session; for continuous recordings, it is calculated separately within each analysis window. Integration uses the trapezoidal rule over the recorded timestamps and is performed separately across contiguous finite portions of the trace so that nonfinite gaps are not bridged.

### Slow-signal analysis

Slow-signal analysis is performed independently of event detection and uses the raw signal and reference channels rather than the corrected Δ*F*/*F* or Δ*F* traces. For each ROI, let $S_{\mathrm{raw}}(t)$ denote the raw signal fluorescence and $R_{\mathrm{raw}}(t)$ the simultaneously recorded raw reference fluorescence. A single recording-wide robust affine signal–reference relationship is estimated as follows:

$$\hat{R}(t)=aR_{\mathrm{raw}}(t)+b,$$

where a and b are the fitted slope and intercept and $\hat{R}(t)$ is the fitted reference. A reference-normalized residual is then calculated as follows:

$$D(t)=\;\frac{S_{\mathrm{raw}}(t)-\hat{R}(t)}{\hat{R}(t)}.$$

For each intermittent recording session or continuous analysis window k, the slow-signal value $T_{k}$ is defined as the second percentile of the finite D(t) values within that interval:

$$T_{k}=\;P_{2}\{D(t):t\in k\}.$$

This statistic provides a low-quantile summary of the reference-normalized signal within each session or analysis window rather than a low-pass-filtered trace. Primary slow-signal values are expressed as fractional $\Delta F/F_{0}$, without percentage scaling.

For intermittent recordings, the recording-wide signal-reference fit is estimated once across the loaded recording sessions for each ROI, after which the second-percentile value is calculated separately within each session. For continuous recordings, the fit is likewise estimated across the full recording, and the resulting residual is summarized within nonoverlapping analysis windows. The default continuous analysis window is 600 s. Window boundaries determine where slow-signal summaries are calculated but do not reset the recording-wide signal-reference fit.

The recording-wide robust fit is initialized by ordinary least squares and refined over three iterations using residual median/MAD scaling, retaining samples with residuals within four times the robust MAD-based scale. If the recording-wide fit cannot be estimated, has a nonpositive slope, or produces no valid primary output for an ROI, the slow-signal summary is instead derived from the raw signal alone. For each session or analysis window k, the second-percentile raw signal value is defined as follows:

$$B_{k}=\;P_{2}\{S_{\mathrm{raw}}(t):t\in k\}.$$

The sequence of $B_{k}$ values is fit with the bounded exponential:

$$E(t)=c+Ae^{-t/\tau },$$

where c is the asymptotic offset, A is the exponential amplitude, and τ is the decay time constant. The bleach-corrected fallback value is then as follows:

$$T_{k}=\;B_{k}-E(t_{k})+\mathrm{mean}(E),$$

where $t_{k}$ is the session time for intermittent recordings or the analysis-window midpoint for continuous recordings. Fallback values are reported in raw fluorescence arbitrary units.

### Code accessibility

The code and software described in the paper are freely available online at https://github.com/jones-lab-tamu/long-term-photometry. The code is available as Extended Data.

10.1523/ENEURO.0128-26.2026.d1 Data 1 Code and software described in this paper. Download Data 1, ZIP file.

## Results

### A guided workflow for long-term photometry analysis

We organized the software around a guided workflow that structures the major decisions involved in setting up, executing, and reviewing a long-term photometry analysis (Fig. 1). Users can begin a new analysis or reopen results from a previously completed analysis. For a new analysis, the user defines the data source, recording structure, included ROIs, and timing information before proceeding to correction and feature detection. The user then previews and confirms a correction strategy and configures and previews feature-detection settings for each included ROI before reviewing the complete analysis plan and initiating the run. After the run is complete, the application provides a Review workspace for examining correction performance, slow-signal structure, corrected signals, and event-analysis results.

**Figure 1.**
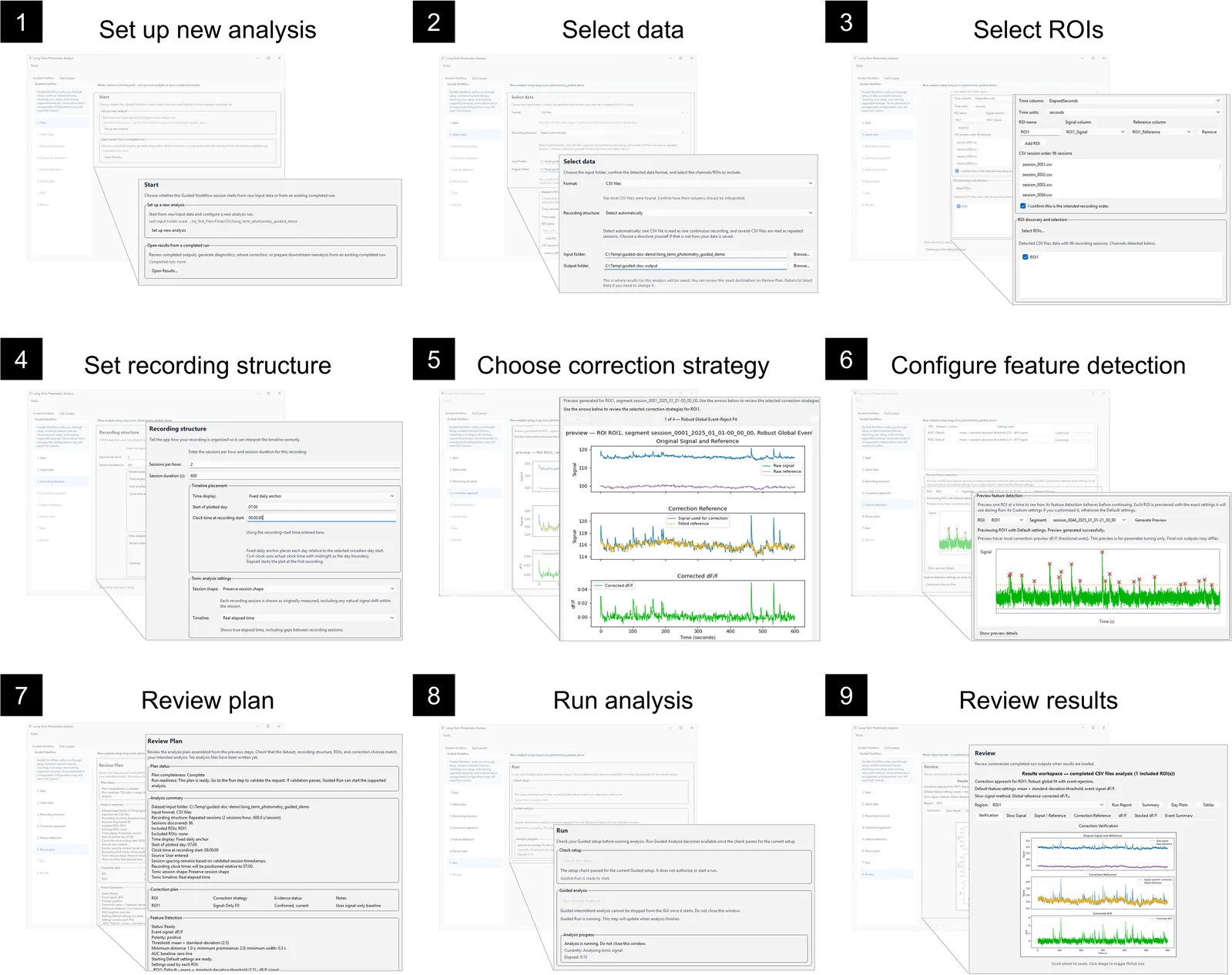
Guided workflow for long-term photometry analysis. The guided workflow organizes analysis into a nine-step workflow that leads the user from analysis setup through review of completed results. (**1**) The user starts a new analysis or opens results from a previously completed analysis. (**2**) The recording structure (continuous or intermittent), input format (RWD, Neurophotometrics, or CSV), input data, and output location are selected. (**3**) Available ROIs are identified and included in or excluded from the analysis. (**4**) The recording structure is defined, including session timing and timeline alignment. (**5**) A correction strategy is selected, previewed, and confirmed for each included ROI. (**6**) Feature-detection settings are configured and previewed for each included ROI. (**7**) The complete analysis plan is reviewed. (**8**) The reviewed plan undergoes a setup check and is then executed as a single analysis run. (**9**) Completed runs are reviewed within the application, allowing the user to inspect correction performance, slow-signal structure, corrected signals, and event-analysis results as applicable.

The software supports both intermittent recordings collected as repeated sessions and continuous recordings collected as a single uninterrupted time series. To provide examples of both recording structures, the application includes a built-in generator for intermittent and continuous synthetic demonstration datasets (Fig. 2). The intermittent dataset is organized as repeated 10 min recording sessions distributed across 48 h, whereas the continuous dataset contains an uninterrupted 48 h recording. Both synthetic datasets contain signal and reference channels that share noise and low-frequency fluctuations, while GCaMP-like transients are restricted to the signal channel. The generator also imposes 24 h rhythms in slow signal and event frequency, with the peak in event frequency aligned to the peak of the slow signal.

**Figure 2.**
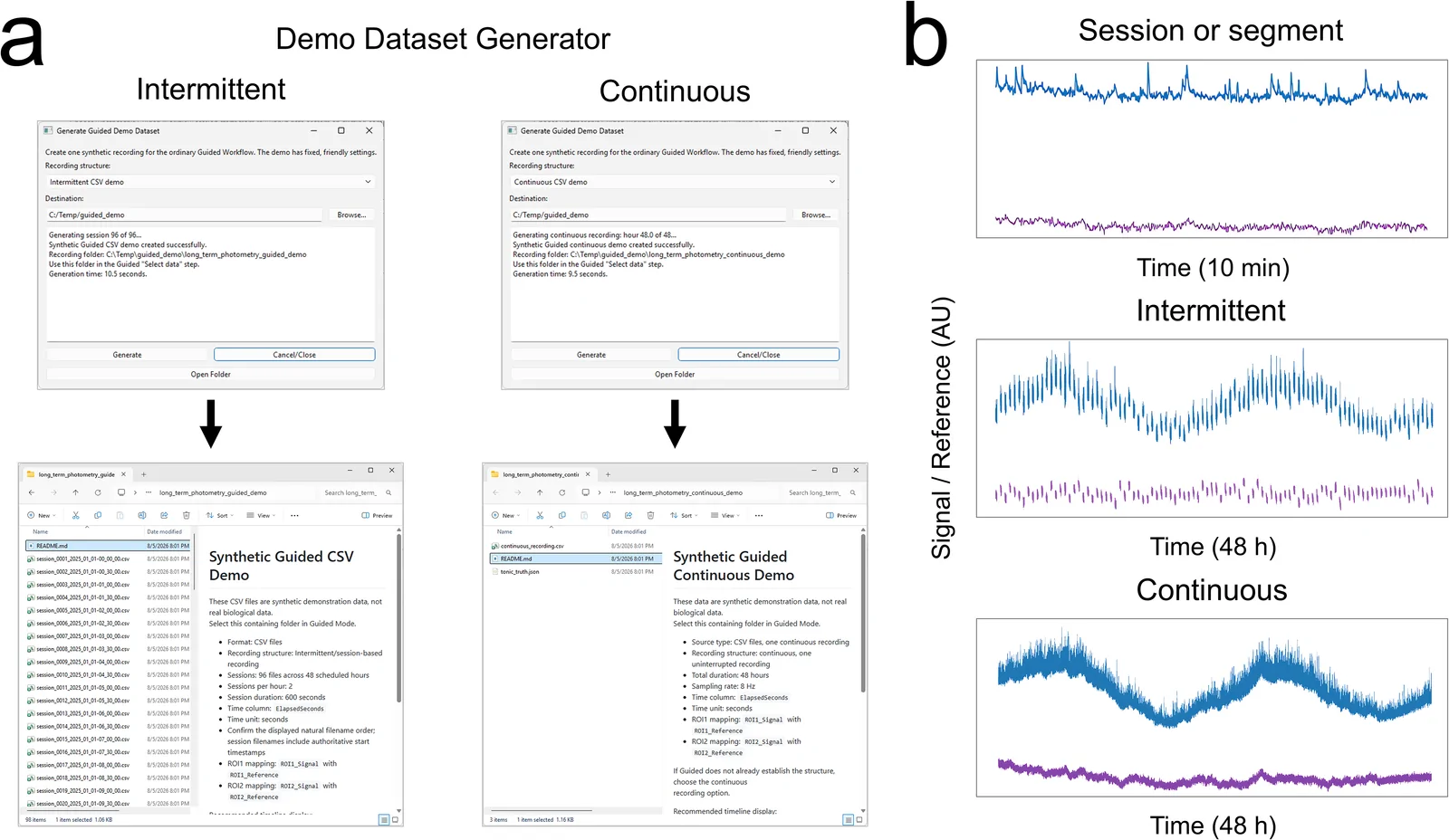
Synthetic demonstration datasets for intermittent and continuous recordings. ***a***, The built-in demo dataset generator creates synthetic intermittent or continuous datasets for use with the guided workflow. Each dataset includes the required data files and a README describing the recording structure, timing, and channel assignments. ***b***, Representative signal and reference traces from the generated datasets. Top, A representative 10 min session from an intermittent dataset or segment from a continuous dataset. Within these 10 min intervals, the signal (blue) and reference (purple) channels share noise and low-frequency fluctuations, while GCaMP-like transients occur only in the signal channel. Middle, An intermittent dataset containing repeated 10 min sessions distributed across a 48 h recording. Bottom, A continuous dataset containing uninterrupted signal and reference channels across 48 h. The generator imposes a 24 h rhythm on both the slow signal and event frequency, with event frequency peaking at the maximum of the slow signal. The datasets are provided for tutorial use and workflow testing.

### Correction strategy is an explicit, ROI-specific analytical decision

Signal correction is performed separately for each included ROI, allowing the correction strategy to be selected according to the behavior of the signal and reference in that ROI rather than imposed uniformly across all ROIs (Fig. 3*a*). Before the final analysis, the user can compare candidate correction strategies on a selected recording segment and confirm the strategy to be used for each ROI. The preview is used to compare candidate strategies on the selected segment; the fitted model from that preview is not carried forward into the final analysis. Once a strategy is selected, correction is re-estimated from the production data rather than reusing the model fitted to the preview segment. For intermittent recordings, correction models are estimated independently for each recording session, whereas for continuous recordings they are estimated within successive correction segments. Thus, the preview allows candidate strategies to be compared on a selected segment without making that preview fit part of the final analysis.

**Figure 3.**
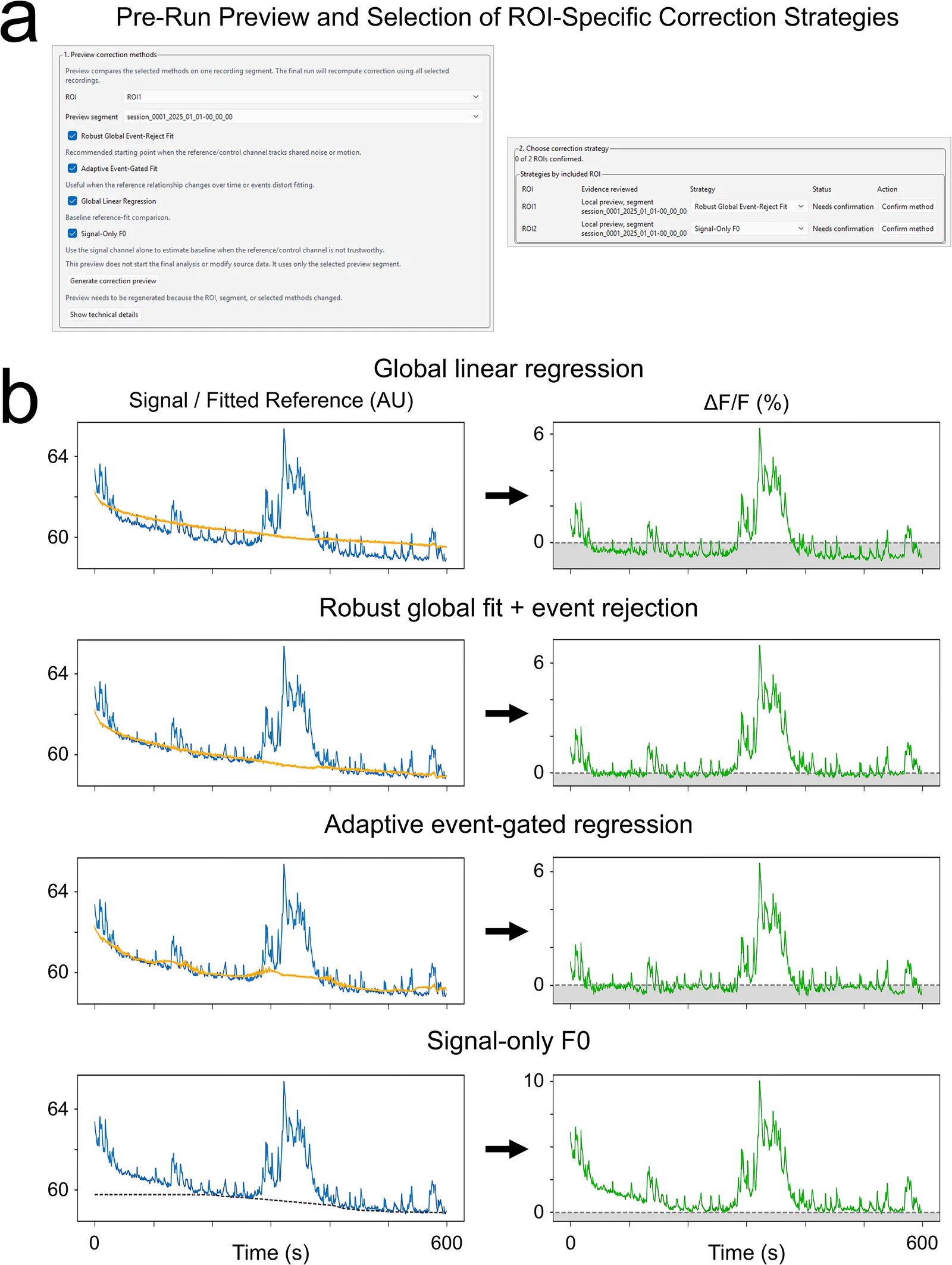
Pre-run selection of ROI-specific correction strategies. ***a***, The guided workflow allows the user to compare candidate correction strategies on a selected recording segment before confirming one strategy for each included ROI. ***b***, Four correction strategies applied to the same 10 min recording segment. For each strategy, the left panel shows the signal (blue) and fitted reference (yellow) or the signal-derived F0 baseline for Signal-Only F0 (dashed line), and the right panel shows the resulting Δ*F*/*F* trace (green). Global linear regression estimates a single signal–reference relationship using all samples within the fitted session or correction segment, allowing the large transient to influence the fit and mis-center much of the corrected trace below zero. Robust global fitting with event rejection iteratively excludes samples with large residuals or high local variance while retaining a single relationship across the fitted interval. Adaptive event-gated regression allows the fitted reference to change gradually over time but updates the time-varying signal–reference relationship only from samples that pass the event-gating and trust criteria. Signal-only F0 does not use the reference channel and instead estimates a slowly varying baseline directly from the signal.

The available correction strategies differ in how they estimate the correction reference and in the assumptions they make about the stability of the signal–reference relationship (Fig. 3*b*). Global linear regression estimates a single relationship from all samples within the current session or correction segment, allowing large signal-specific transients to influence the fit. In the example shown, the large transient biases this fit and shifts much of the resulting Δ*F*/*F* below zero, illustrating why a global fit based on all samples within the fitted interval can be inadequate when prominent signal-specific transients are present. Robust global fitting with event rejection also retains a single relationship within the current session or correction segment but iteratively excludes samples with large residuals or high local variance. Adaptive event-gated regression allows the fitted relationship to change gradually over time, with updates restricted to samples that pass event-gating and trust criteria. Signal-only F0 does not use the reference channel and instead estimates a slowly varying baseline directly from the signal.

Correction becomes more challenging when the signal-reference relationship changes over time or when biologically relevant changes in the signal are not tracked by the reference channel (Fig. 4). In a representative example of a changing signal-reference relationship, robust global fitting imposed a single relationship across the segment, causing the fitted reference to exceed the signal during one portion of the recording and fall below it near the end (Fig. 4*a*). This produced an artificial upward curvature in the corrected Δ*F*/*F*. Adaptive event-gated regression was able to accommodate the changing signal-reference relationship across the same segment, maintaining a better alignment of the fitted reference with the signal and removing the resulting distortion. In a separate example in which prolonged signal elevations were not tracked by the reference channel, the elevations pulled the robust global fit upward, causing the fitted reference to exceed the signal through much of the segment and shifting the corrected Δ*F*/*F* below zero (Fig. 4*b*). Signal-only F0 avoids that distortion because its baseline estimate is not driven by the poorly matched reference, allowing the prolonged elevations to remain above baseline. These examples show why the workflow treats correction strategy as an ROI-specific analytical decision rather than applying a single correction strategy by default.

**Figure 4.**
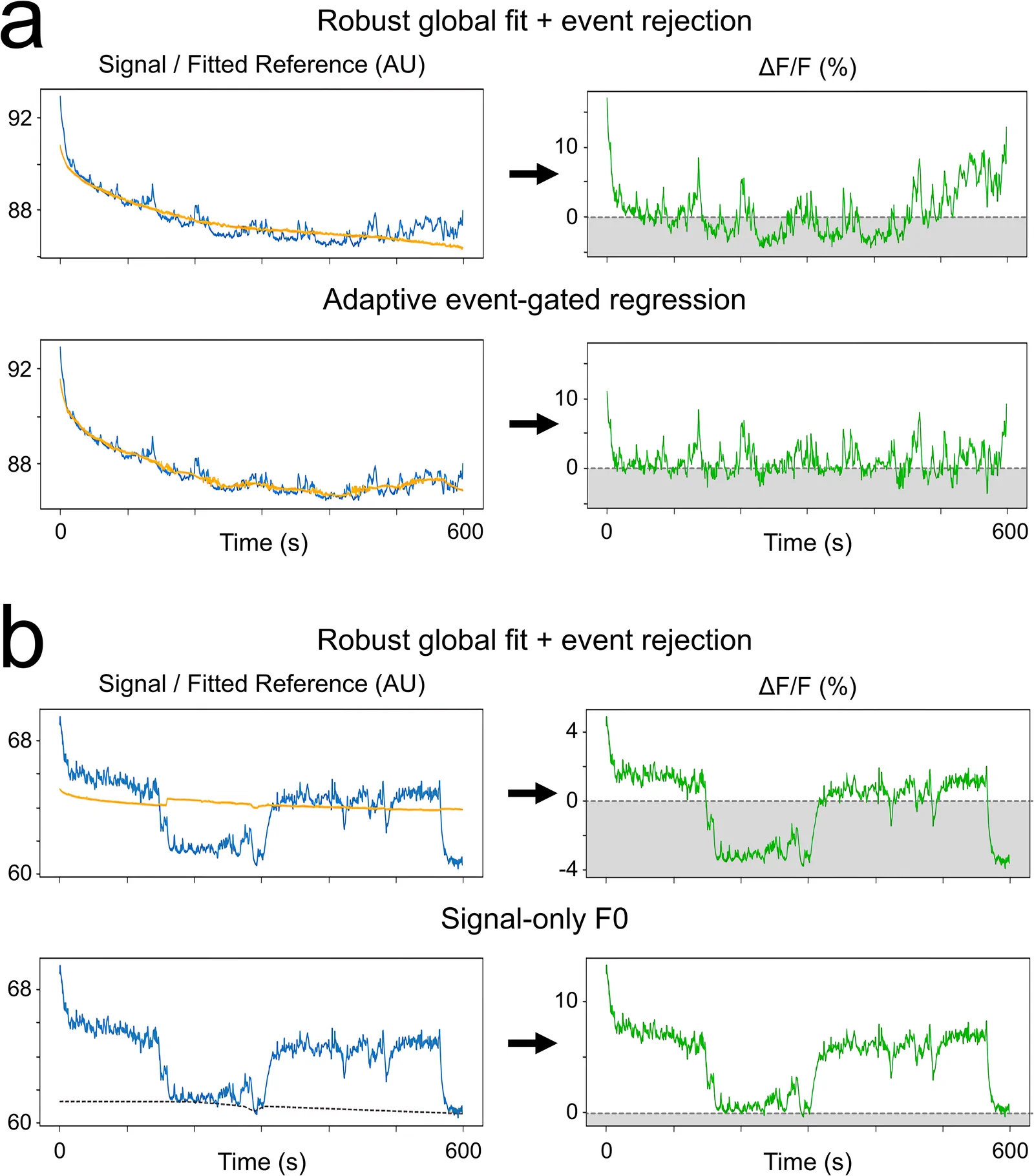
Correction strategies for recordings with changing or unreliable signal-reference relationships. ***a***, Comparison of robust global fitting with event rejection and adaptive event-gated regression for a segment in which the signal-reference relationship changes over time. With robust global fitting, one signal-reference relationship is estimated across the segment. The fitted reference exceeds the signal during one portion of the segment and falls below it near the end, producing an artificial upward curvature in the corrected Δ*F*/*F*. Adaptive event-gated regression allows the fitted relationship to change across the segment and removes this distortion. ***b***, Comparison of robust global fitting with event rejection and signal-only F0 for a segment containing prolonged signal elevations that are not tracked by the reference channel. The prolonged signal elevations pull the robust global fit upward, causing the fitted reference to exceed the signal through much of the segment and shifting the resulting Δ*F*/*F* below zero. Signal-only F0 estimates a slowly varying baseline from the signal itself, allowing the prolonged signal elevations to remain above baseline. Left, Signal, blue; fitted reference for robust global and adaptive methods, yellow; signal-derived F0 baseline for signal-only F0, dashed line. Right, Δ*F*/*F*, green.

### Event-detection criteria are configured downstream of signal correction

Event detection is configured after signal correction, so changes in detection criteria alter which excursions are classified as events without changing the corrected Δ*F*/*F* trace (Fig. 5*a*). For each included ROI, the user can retain the shared default settings or define ROI-specific parameters and preview their effects on a selected recording segment before confirming the settings used for analysis. Detection is configured by specifying signal polarity and a threshold method (mean, median, percentile, or absolute) and value. Additional criteria, including minimum peak distance, prominence, and width, further constrain which excursions are detected. Optional prefiltering can also be applied before detection. The preview overlays detected events on the corrected trace, allowing the consequences of parameter changes to be inspected before the final analysis.

**Figure 5.**
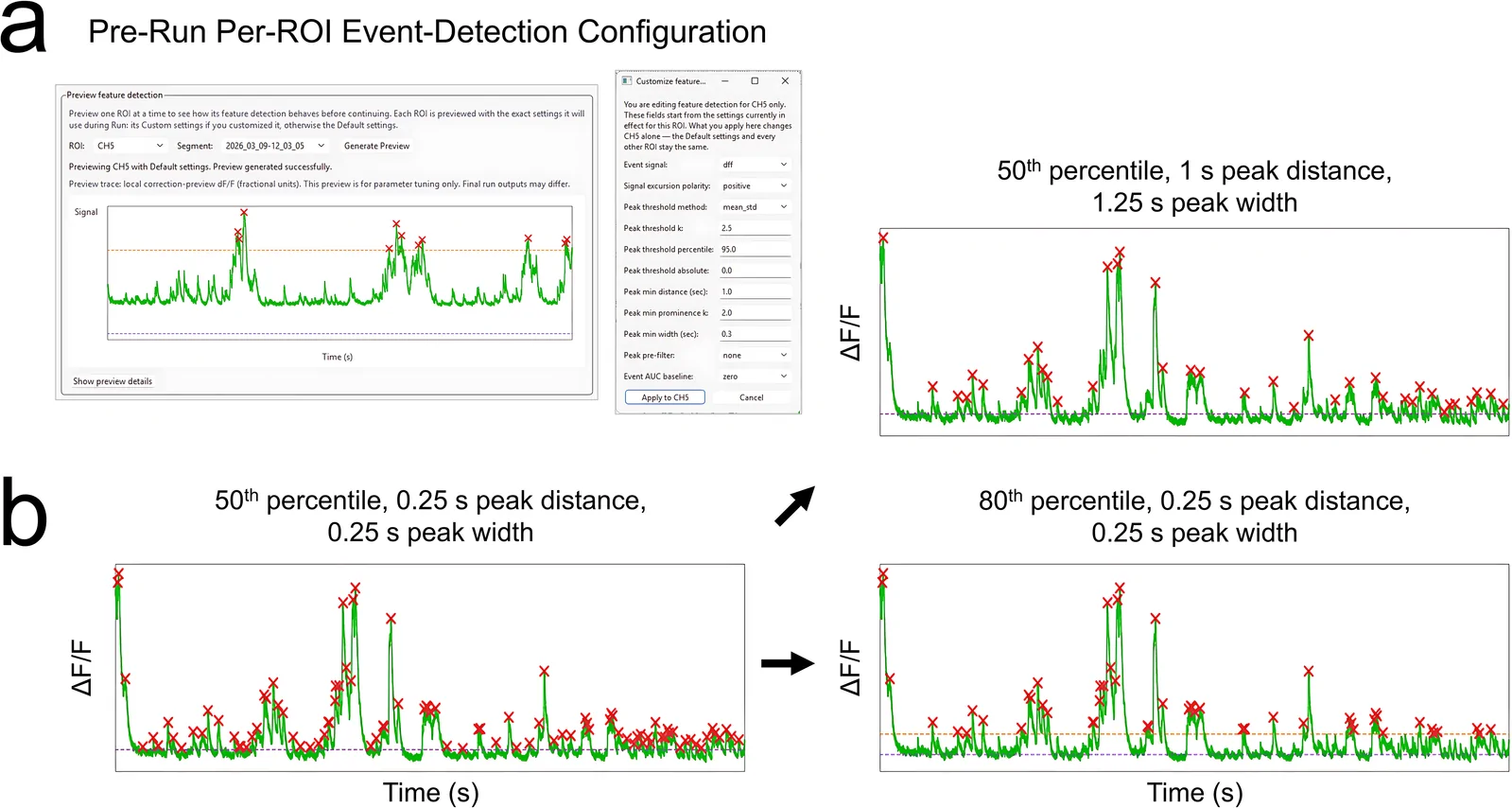
Pre-run configuration of ROI-specific event-detection settings. ***a***, The guided workflow allows the user to preview event detection for a selected ROI and recording segment before confirming the settings used for analysis. Detection parameters can retain the shared default settings or be customized for an individual ROI. The preview displays the corrected Δ*F*/*F* trace together with events identified under the selected settings, allowing the effects of parameter changes to be inspected before the production run. ***b***, Representative effects of changing event-detection parameters on the same corrected Δ*F*/*F* trace. With a 50th-percentile threshold, 0.25 s minimum peak distance, and 0.25 s minimum peak width, numerous lower-amplitude excursions satisfy the detection criteria. Increasing the threshold to the 80th percentile restricts detection to larger-amplitude excursions. Together, increasing the minimum peak distance to 1 s and the minimum peak width to 1.25 s further restricts detection to more widely separated and sustained excursions. Red crosses indicate detected peaks; the dashed orange line indicates the upper detection threshold.

In a representative example, three different parameter sets were applied to a single corrected Δ*F*/*F* trace (Fig. 5*b*). Highly permissive settings (a 50th-percentile threshold with a 0.25 s minimum peak distance and width) identified numerous lower-amplitude excursions. Keeping the distance and width criteria unchanged while increasing the threshold to the 80th percentile restricted detection primarily to larger-amplitude excursions. Alternatively, retaining the 50th-percentile threshold while increasing the minimum peak distance to 1 s and the minimum peak width to 1.25 s restricted detection to more widely separated and sustained excursions. Together, these examples show that different classes of event-detection parameters can alter detection selectivity in different ways.

### Completed analyses can be inspected across session-level and multiday timescales

After analysis, correction performance can be inspected for each included ROI in the Review workspace (Fig. 6*a*). For intermittent recordings, individual recording sessions remain positioned according to their time of day rather than being concatenated into a continuous trace. Across a representative 24 h interval with two 10 min sessions per hour, the raw signal and reference, fitted correction reference, and corrected Δ*F*/*F* can be compared across sessions while preserving each session's time-of-day position (Fig. 6*b*).

**Figure 6.**
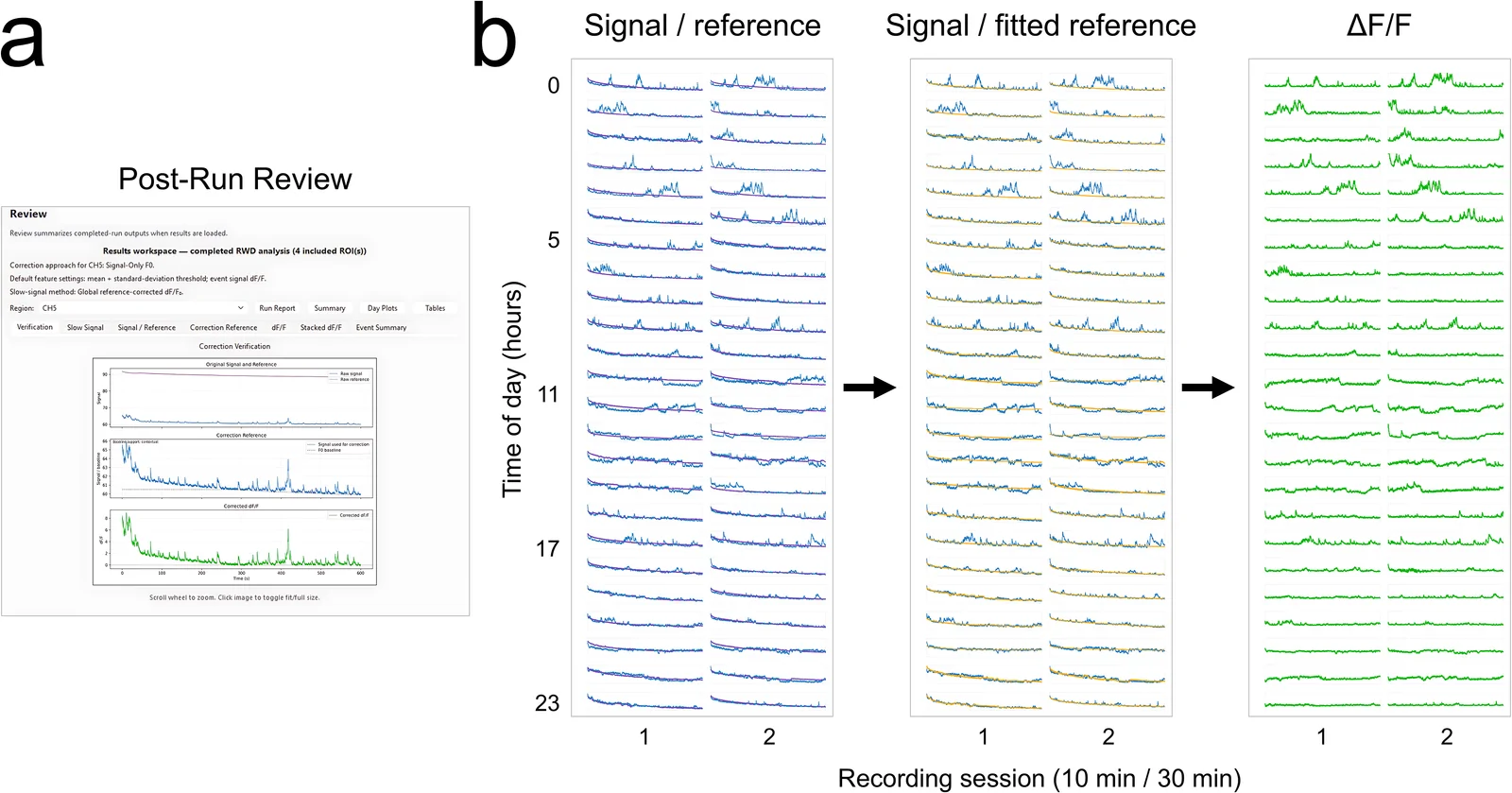
Post-run review of correction across the recording timeline. ***a***, After an analysis is completed, the guided workflow provides a Review workspace for inspection of the saved results for each included ROI. The example shown here is the Verification tab, which displays representative correction outputs and identifies the correction strategy used; additional tabs provide access to other saved result views. ***b***, Example outputs from a representative 24 h interval extracted from a longer intermittent recording collected as two 10 min sessions per hour. Left, Raw signal (blue) and reference (purple) traces for each recording session. Middle, Raw signal (blue) overlaid with the fitted correction reference (yellow) used for Δ*F*/*F* calculation. Right, The resulting Δ*F*/*F* traces (green). Sessions are arranged according to time of day rather than concatenated, preserving the temporal organization of the recording while allowing the fitted correction and corrected signal to be inspected across individual sessions.

A representative 12 d synthetic recording demonstrates how long-duration photometry data can be viewed as session-resolved traces, longitudinal summaries, and slow-signal structure (Fig. 7). Day-aligned stacked Δ*F*/*F* traces preserved within-session fluorescence dynamics across successive days (Fig. 7*a*), whereas session-level analyses of integrated fluorescence and event frequency provided quantitative measures that could be followed across the full recording (Fig. 7*b*,*c*). A separate slow-signal analysis captures slow, sustained fluorescence fluctuations distinct from transient event-related activity. Synthetic datasets with similar transient event-related activity but differing slow-signal amplitudes produced correspondingly different slow Δ*F*/*F* profiles (Fig. 7*d*,*e*).

**Figure 7.**
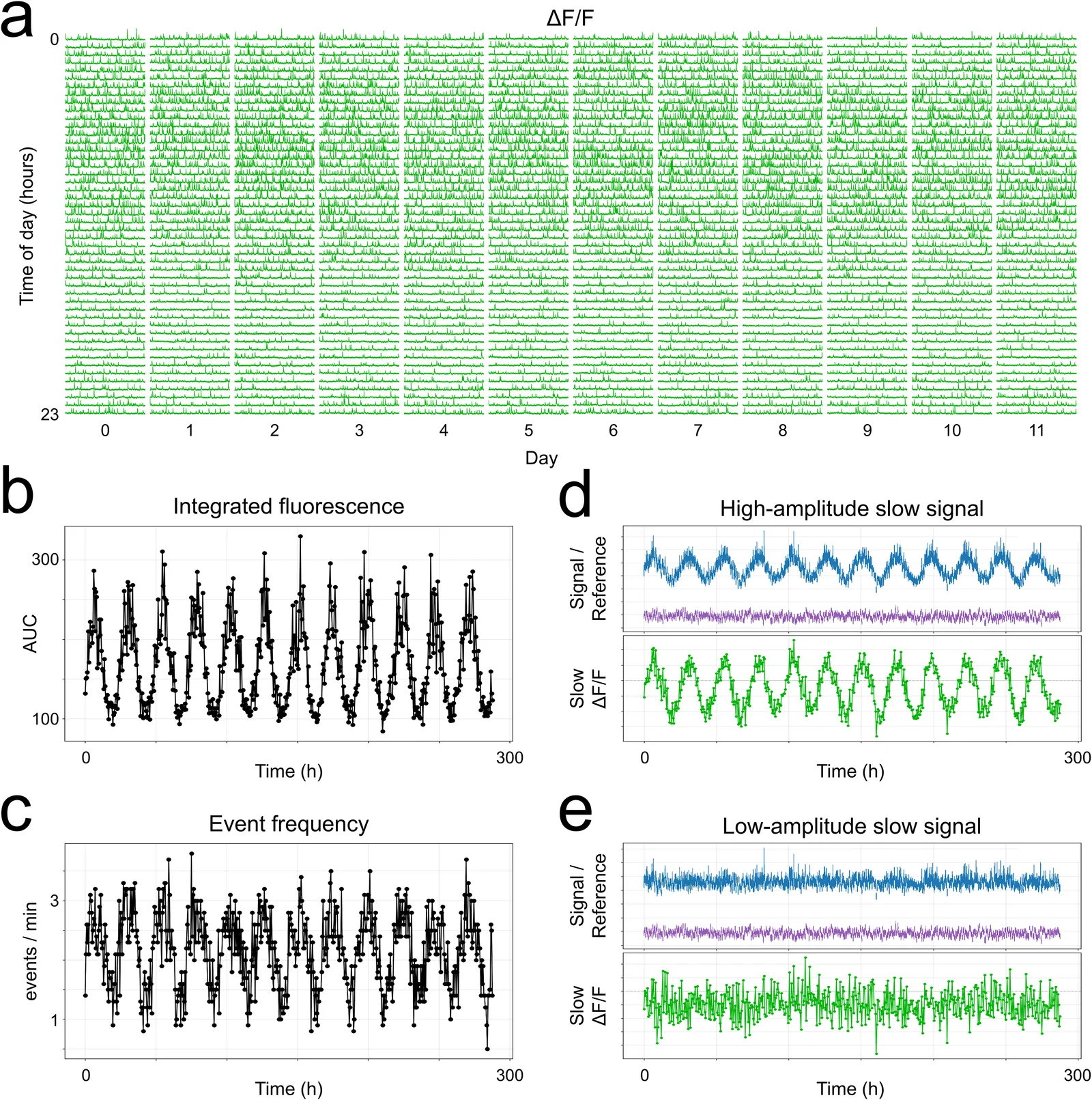
Long-duration visualization, signal summaries, and slow-signal outputs. ***a***, Day-aligned stacked Δ*F*/*F* traces from a 12 d synthetic demonstration dataset containing two 10 min recording sessions per hour. Individual sessions are arranged according to time of day within each plotted day. ***b***, Integrated fluorescence across the full recording, calculated separately for each 10 min recording session as the area of the corrected Δ*F*/*F* trace above zero. ***c***, Event frequency across the full recording, calculated separately for each 10 min recording session as the number of detected events per minute. ***d***, Example synthetic dataset with a high-amplitude slow signal. Top, Signal (blue) and reference (purple) fluorescence across the recording. Bottom, The resulting slow Δ*F*/*F* output (green). ***e***, Example synthetic dataset generated with a low-amplitude slow signal, shown in the same format as panel ***d*** to illustrate the resulting weaker slow-signal output.

### Long-term photometry analysis across sensor classes

To examine the applicability of the workflow beyond calcium photometry, we analyzed long-term recordings obtained with genetically encoded sensors for calcium, acetylcholine, and dopamine (Fig. 8). The same long-term analysis framework was applied to GCaMP7s, GRAB-ACh4m, and GRAB-DA3m recordings, producing day-aligned Δ*F*/*F* traces across all three sensor classes.

**Figure 8.**
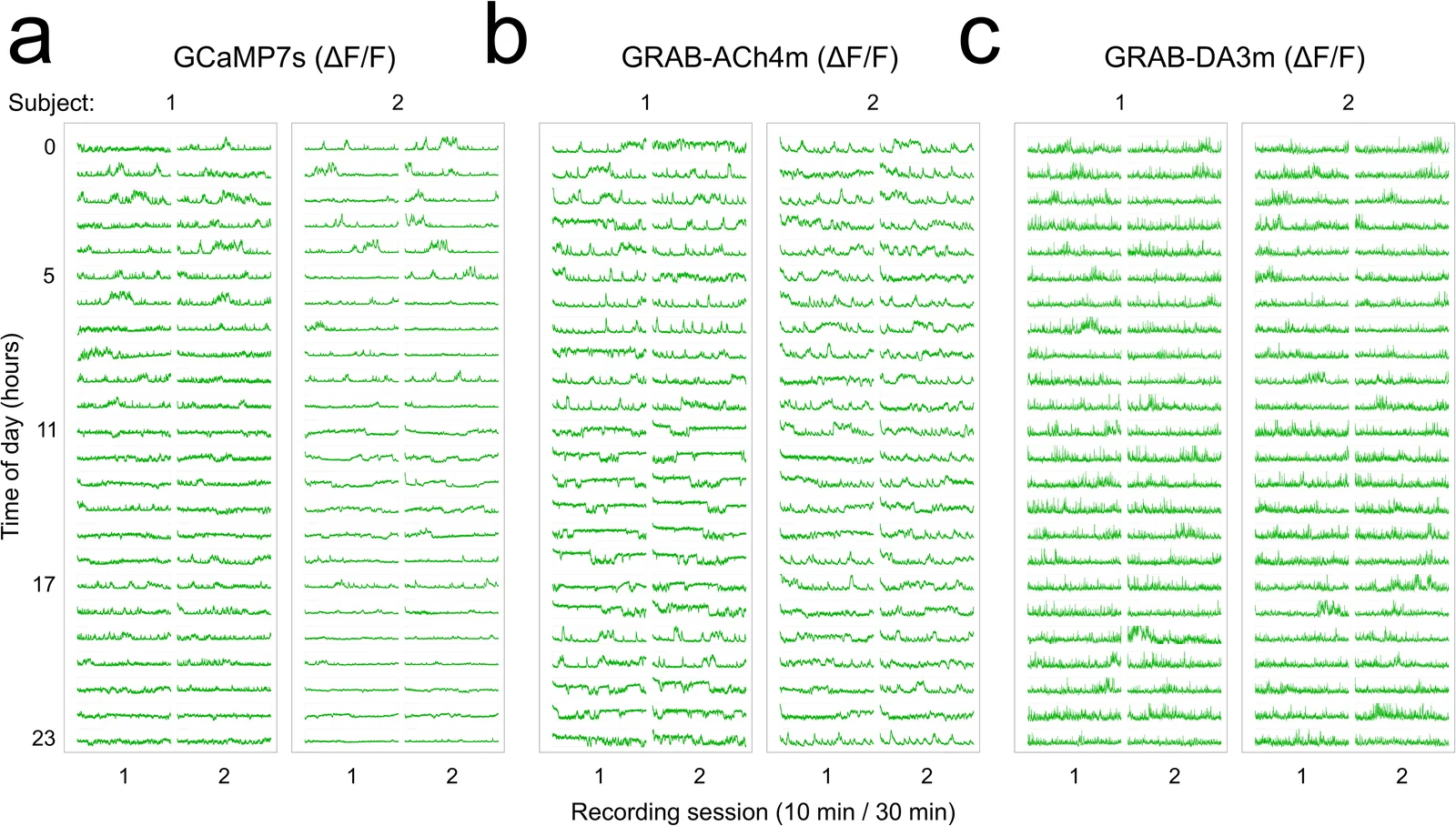
Long-term photometry analysis across sensor classes. Day-aligned Δ*F*/*F* traces from two representative animals for each of three genetically encoded fluorescent sensors measuring calcium, acetylcholine, or dopamine, shown over 24 h with two 10 min recording sessions per hour. For each subject, recording sessions are arranged according to time of day. ***a***, GCaMP7s recordings from the medial septum. ***b***, GRAB-ACh4m recordings from the hippocampus. ***c***, GRAB-DA3m recordings from the nucleus accumbens.

## Discussion

Long-term photometry changes the practical demands of analysis as recordings extend across days. Such datasets can contain hundreds or thousands of individual sessions or long uninterrupted time series, making exhaustive trace-by-trace inspection impractical. Existing open-source photometry tools provide important capabilities for signal processing and event-based analysis (Bruno et al., 2020; Sherathiya et al., 2021; Conlisk et al., 2023; Keevers and Jean-Richard-Dit-Bressel, 2025), but long-duration recordings place additional demands on temporal organization, scalable review, and the handling of analytical decisions across large datasets. At this scale, reproducible analysis requires consequential choices to be explicit, reviewable, and recorded as part of a defined analysis plan. The guided workflow presented here addresses this problem by structuring correction and event-detection choices before analysis and allowing their consequences to be inspected afterward.

Among these analytical choices, correction is especially consequential because it directly determines the corrected Δ*F*/*F* used for downstream analysis. The examples in Figures 3 and 4 show that correction performance depends on the structure and stability of the signal-reference relationship and that no single strategy is appropriate across all recording conditions. Reference-based correction can reduce fluctuations shared between signal and reference, but it is less informative for signal-specific excursions whose origin cannot be resolved from the fluorescence traces alone. Correction therefore remains a matter of analytical judgment rather than a decision that can be resolved automatically. The guided workflow constrains and records that judgment rather than attempting to eliminate it.

Event detection introduces a separate layer of analytical judgment downstream of correction. Unlike correction, which changes the Δ*F*/*F* trace itself, event-detection settings determine which excursions in the corrected trace are classified as events. Figure 5 illustrates that distinct classes of detection parameters alter event classification in different ways, and there is no single parameter set that can be assumed to be appropriate across all recordings. Appropriate criteria will depend on the sensor, circuit, and recording conditions because fluorescence transients can vary substantially in amplitude, duration, and temporal structure across experimental contexts. Event detection should therefore be treated as a defined analytical choice rather than as a property inherent to the corrected trace. The guided workflow makes these criteria explicit and allows them to be specified separately for each ROI before analysis.

Event-related summaries, however, capture only one aspect of a long-duration photometry recording. Session-resolved traces preserve within-session fluorescence dynamics, whereas longitudinal summaries make changes in signal and event measures easier to follow across days. Slow-signal analysis provides a complementary representation of the recording by describing sustained fluorescence structure independently of event-based summaries. This distinction reflects prior work emphasizing that slow fluorescence variation and transient activity can carry different information and should not be conflated within a single summary measure (Tang et al., 2025).

The present examples also broaden the demonstrated scope of the workflow beyond calcium photometry to long-term recordings obtained with acetylcholine and dopamine sensors (Fig. 8). Across these sensor classes, the ability to tailor correction and event-detection settings to individual recordings is important because sensor kinetics and biological context affect how fluorescence changes should be interpreted. That variability reinforces the importance of making analytical choices explicit, both to support reproducible analysis and to interpret their consequences in the context of each recording.

## Synthesis

Reviewing Editor: Jibran Khokhar, Western University Department of Anatomy and Cell Biology Decisions are customarily a result of the Reviewing Editor and the peer reviewers coming together and discussing their recommendations until a consensus is reached. When revisions are invited, a fact-based synthesis statement explaining their decision and outlining what is needed to prepare a revision will be listed below. The following reviewer(s) agreed to reveal their identity: Deniz Atasoy, Alessandra Porcu.

This manuscript presents a timely and potentially valuable software framework for the analysis of long-term fiber photometry recordings, addressing an important methodological gap in the field. The focus on standardizing and streamlining analyses across recordings spanning hours to days is particularly relevant, as long-term datasets pose distinct analytical challenges compared to conventional short-duration recordings. The inclusion of visualization tools within the workflow is a notable strength, as it allows users to inspect fitting, detrending, and correction procedures and reduces the likelihood of inappropriate preprocessing substantially altering data interpretation. The framework also appears promising for applications involving circadian dynamics, behavioral state transitions, and prolonged neural monitoring.

Major Comments

Insufficient validation across datasets, sensors, and recording conditions

The representative analyses appear to rely on recordings from only two mice using GCaMP7s. This makes it difficult to determine how the software performs with weaker signals, substantial bleaching, motion artifacts, noisy recordings, or alternative sensor types. The manuscript would be strengthened by including validation using additional datasets, either from other laboratories or publicly available recordings, encompassing different sensors and experimental paradigms. Alternatively, the authors should clearly state that the current framework was specifically optimized for GCaMP7s recordings.

Need for clarification regarding artifact handling

The manuscript does not sufficiently describe how motion artifacts, head movement, or other non-biological fluctuations are identified and corrected. Because artifact correction is central to reliable fiber photometry analysis, the workflow for handling these issues should be described in detail.

Isosbestic correction should be presented as a core preprocessing step

The categorization of isosbestic correction within an "advanced" workflow branch is conceptually concerning. Isosbestic correction is generally considered a standard preprocessing procedure required prior to downstream analyses because it accounts for motion artifacts, bleaching, and nonspecific signal fluctuations. The manuscript should clarify that the correction itself is part of the standard workflow, with only optional parameter tuning categorized as advanced.

Potential analytical bias introduced by post hoc correction retuning

The rationale and safeguards surrounding "retuning" of correction procedures require further clarification. While different fitting approaches can substantially influence corrected ΔF/F signals, especially during large transients, iterative post hoc adjustment risks introducing analytical bias or overfitting. The authors should clearly define:

-Under what circumstances retuning is appropriate

-How correction methods are objectively selected

-Whether quantitative metrics guide optimization

-How user-dependent bias is minimized

Insufficient detail regarding event detection methodology

The event reanalysis workflow is conceptually clearer because upstream correction remains fixed while detection parameters are adjusted. However, the manuscript does not adequately describe how events are identified. The authors should specify whether detection relies on fixed thresholds, adaptive thresholds, prominence measures, signal-to-noise criteria, or other algorithms, and explain how reproducibility is maintained across users and datasets.

Need for clarification of tonic/phasic decomposition across sensor classes

The manuscript should clarify whether the tonic/phasic separation framework is intended to be sensor-agnostic. Different calcium and neurotransmitter sensors exhibit substantially different kinetics and baseline characteristics, which may affect the criteria used to separate tonic versus phasic activity. The authors should discuss how the workflow accommodates sensor-specific dynamics and whether parameter adjustment is required across sensor types.

Insufficient description of software functionality and workflow

The manuscript currently provides limited explanation of the software interface, analytical options, and processing workflow. It is unclear whether the software includes integrated documentation, tutorials, or explanatory guidance for users. More comprehensive methodological detail is necessary to evaluate reproducibility and usability.

While the interface includes hover-over information prompts, these are currently too limited to guide users unfamiliar with the underlying analytical procedures. If the goal is to standardize and streamline fiber photometry analysis across users with varying levels of computational expertise, a detailed step-by-step tutorial and more comprehensive help resources are needed, particularly for preprocessing steps such as normalization, detrending, fitting, and correction parameter selection.

Clarification needed regarding handling of long continuous recordings

Testing with a multi-day continuous recording resulted in only the first 10 minutes being displayed upon upload. This raises an important question regarding whether the software is optimized for continuous recordings spanning hours to days, as described in the manuscript, or primarily for segmented/time-lapse acquisition paradigms. If continuous recordings are supported, the authors should clarify how these are loaded, visualized, and tuned. If additional settings are required, this should be explained in the documentation. If only time-lapse data are currently supported, that limitation should be clearly stated.

Need for comprehensive user documentation and tutorial materials

A major opportunity for improvement is documentation. The app is installable and usable without Python expertise, and basic upload is straightforward, which is a strength. However, users with little programming or signal-processing experience would benefit from a much more detailed step-by-step tutorial. The current hover-over information prompts are too limited to explain the rationale behind the many available options. More extensive guidance, ideally including example datasets, walkthroughs, and troubleshooting documentation, would make the software significantly more accessible and reproducible across users.

Minor Comments

Clarify file compatibility and supported acquisition systems

Figure 2 suggests compatibility primarily with .rwd and .npm formats. The manuscript should explicitly state:

-Which acquisition systems are supported

-Whether Neurophotometrics, Tucker Davis, Doric, and other common systems are compatible

-Whether generic .csv or .txt files can be imported

-Clarify handling of multiple files and batch processing

Additional detail is needed regarding:

-Import of multiple sequential recording files

-Batch processing across animals

-Simultaneous visualization of multiple recordings

-Standardization of tuning across datasets

-Clarify scalability of event rejection workflows

The manuscript should explain whether artifact/event rejection must be performed individually for each segment or whether session-wide correction is possible for very long recordings containing many large events.

Provide more detail regarding tuning options shown in Figure 5

The manuscript should define the tuning parameters, explain what each adjustment accomplishes, and clarify whether standardized presets or recommended settings exist.

Clarify the "dynamic fit" procedure

The functionality and rationale underlying the "dynamic fit" approach are not sufficiently explained. More methodological detail would improve interpretability and reproducibility.

Clarify expected user expertise requirements

It is unclear whether users are expected to understand the underlying analytical methods in detail or whether the software provides guidance sufficient for less experienced users.

Clarify software outputs and export capabilities

The manuscript should specify:

Output file formats

Whether intermediate processing stages can be exported

Compatibility with downstream statistical analysis workflows

Consider expanding discussion of generalizability and limitations

The discussion would benefit from a more explicit consideration of the software's current limitations, including potential constraints related to sensor specificity, recording conditions, and user-dependent parameter optimization.

## Author Response

This manuscript presents a timely and potentially valuable software framework for the analysis of long-term fiber photometry recordings, addressing an important methodological gap in the field. The focus on standardizing and streamlining analyses across recordings spanning hours to days is particularly relevant, as long-term datasets pose distinct analytical challenges compared to conventional short-duration recordings. The inclusion of visualization tools within the workflow is a notable strength, as it allows users to inspect fitting, detrending, and correction procedures and reduces the likelihood of inappropriate preprocessing substantially altering data interpretation. The framework also appears promising for applications involving circadian dynamics, behavioral state transitions, and prolonged neural monitoring.

Major Comments 1) Insufficient validation across datasets, sensors, and recording conditions.

The representative analyses appear to rely on recordings from only two mice using GCaMP7s. This makes it difficult to determine how the software performs with weaker signals, substantial bleaching, motion artifacts, noisy recordings, or alternative sensor types. The manuscript would be strengthened by including validation using additional datasets, either from other laboratories or publicly available recordings, encompassing different sensors and experimental paradigms. Alternatively, the authors should clearly state that the current framework was specifically optimized for GCaMP7s recordings.

We agree that the original manuscript relied too heavily on a single GCaMP7s example and did not adequately demonstrate the applicability of the workflow across different photometry signals and recording conditions. We have substantially expanded the datasets included in the revised manuscript.

The study now includes recordings from six mice spanning three genetically encoded sensors and three brain regions: GCaMP7s recordings from the medial septum, GRAB-ACh4m recordings from the hippocampus, and GRAB-DA3m recordings from the nucleus accumbens. Representative long-term recordings from each sensor class are now shown in Figure 8, demonstrating application of the same analysis workflow to calcium, acetylcholine, and dopamine signals with different signal characteristics.

The revised manuscript also includes synthetic demonstration datasets containing shared signal-reference fluctuations, bleaching, noise, common-mode disturbances, and signal-specific transients. These examples include both intermittent and continuous recording structures as well as different slow-signal amplitudes. Figures 2 and 7 use these datasets to illustrate workflow behavior under defined signal and nuisance conditions.

We also revised the Discussion to clarify that these examples demonstrate applicability across distinct sensor classes and recording characteristics, but do not establish that a single set of analytical parameters is optimal for every sensor or experimental preparation.

2) Need for clarification regarding artifact handling.

The manuscript does not sufficiently describe how motion artifacts, head movement, or other non-biological fluctuations are identified and corrected. Because artifact correction is central to reliable fiber photometry analysis, the workflow for handling these issues should be described in detail.

We agree that the original manuscript did not adequately explain how non-biological fluctuations are handled. We have expanded the Methods and Discussion to clarify the role of the simultaneously recorded reference channel and the available correction strategies.

The software does not use a separate motion-artifact classifier to label individual fluorescence fluctuations as movement-related or other non-biological variation. Instead, the reference-based correction approaches use fluctuations shared between the signal and reference channels to estimate and reduce shared nuisance variation. The revised Methods now describe these correction approaches in detail, including strategies that reduce the influence of large signal-specific excursions during fitting and allow the signal-reference relationship to vary over time.

We also revised the manuscript to make the limitations of this approach explicit. Shared fluctuations captured by the reference channel can be reduced through reference-based correction, but fluorescence data alone generally cannot determine whether a signal-specific excursion reflects neural activity, movement-related variation, or another source of signal change. For the multiday recordings targeted by this software, exhaustive trace-by-trace inspection and manual classification of individual fluctuations are impractical. The workflow therefore emphasizes scalable reference-based correction and transparent inspection of correction performance instead of requiring manual artifact labeling as a routine analysis step.

3) Isosbestic correction should be presented as a core preprocessing step The categorization of isosbestic correction within an "advanced" workflow branch is conceptually concerning. Isosbestic correction is generally considered a standard preprocessing procedure required prior to downstream analyses because it accounts for motion artifacts, bleaching, and nonspecific signal fluctuations. The manuscript should clarify that the correction itself is part of the standard workflow, with only optional parameter tuning categorized as advanced.

We agree. In the revised software and manuscript, reference correction is now a core part of the guided workflow rather than an optional advanced branch. After the recording structure and included ROIs are established, the user evaluates and confirms a correction strategy for each included ROI before configuring event detection or proceeding to analysis. Correction is therefore completed before event detection and downstream signal-summary analyses.

We have also expanded the Methods to describe the available correction approaches and subsequent ΔF/F normalization in detail, and Figures 3 and 4 now illustrate the correction workflow and the consequences of correction choice under different signal-reference conditions.

4) Potential analytical bias introduced by post hoc correction retuning The rationale and safeguards surrounding "retuning" of correction procedures require further clarification. While different fitting approaches can substantially influence corrected ΔF/F signals, especially during large transients, iterative post hoc adjustment risks introducing analytical bias or overfitting. The authors should clearly define: a) under what circumstances retuning is appropriate, b) how correction methods are objectively selected, c) whether quantitative metrics guide optimization, and d) how user-dependent bias is minimized We agree that repeated post hoc adjustment of correction procedures could introduce user-dependent bias, particularly when alternative fits produce meaningful different ΔF/F traces. In response, we substantially revised the software workflow and manuscript. The revised software introduces a guided workflow in which correction strategy is selected before the final analysis is executed, rather than relying on the post-run correction-retuning workflow described in the original manuscript.

In the revised guided workflow, a correction strategy is selected and confirmed separately for each included ROI. The user can compare candidate strategies on a selected recording segment using the signal, fitted reference, and resulting ΔF/F trace. This preview supports comparative inspection of how different strategies model the observed signal-reference relationship and how those choices affect the corrected trace. There is no universal quantitative metric that can establish which correction model is biologically correct across sensors and recording conditions, so the software does not automatically select a correction strategy using a quantitative optimization score.

The revised workflow limits user-dependent bias by separating correction selection from the final analysis and preserving the selected settings with the completed run. The preview fit is not reused in the final analysis; after a strategy is confirmed, the selected method is re-estimated from the full analysis data independently within each intermittent recording session or continuous correction segment. The selected correction strategy and relevant parameters are preserved for each ROI with the completed analysis, making these choices explicit and reviewable.

Figures 3 and 4 now illustrate the pre-run correction-selection workflow and the consequences of correction choice under different signal-reference conditions. The revised Discussion also emphasizes that correction choice remains an analytical judgment and that the guided workflow is intended to constrain, expose, and preserve that judgment rather than imply that a universally optimal correction method can be selected automatically.

5) Insufficient detail regarding event detection methodology The event reanalysis workflow is conceptually clearer because upstream correction remains fixed while detection parameters are adjusted. However, the manuscript does not adequately describe how events are identified. The authors should specify whether detection relies on fixed thresholds, adaptive thresholds, prominence measures, signal-to-noise criteria, or other algorithms, and explain how reproducibility is maintained across users and datasets.

We agree that the original manuscript did not provide enough methodological detail to reproduce the event-detection workflow. We have substantially expanded the Methods to define how events are identified and how detection settings are applied across recordings.

Event detection is performed independently for each ROI using configurable thresholding and peak criteria. Thresholds can be based on the trace mean and standard deviation, median and MAD, user-selected percentiles, or an absolute value. Detected peaks are further constrained by minimum distance, prominence, and width. The revised Methods now define these calculations, polarity handling, optional prefiltering, treatment of nonfinite samples, and default parameter values in detail.

To support reproducibility, the guided workflow provides a common set of default event-detection settings that can be adjusted separately for individual ROIs. The settings used for each ROI are preserved with the completed analysis. Users can preview detection settings on a selected recording segment before execution, but events identified during preview are not retained as final results. Final event detection is performed using the confirmed settings separately within each intermittent recording session or across the reconstructed contiguous corrected trace for continuous recordings.

6) Need for clarification of tonic/phasic decomposition across sensor classes The manuscript should clarify whether the tonic/phasic separation framework is intended to be sensor-agnostic. Different calcium and neurotransmitter sensors exhibit substantially different kinetics and baseline characteristics, which may affect the criteria used to separate tonic versus phasic activity. The authors should discuss how the workflow accommodates sensor-specific dynamics and whether parameter adjustment is required across sensor types.

We agree that the original manuscript did not sufficiently account for differences in sensor kinetics and baseline characteristics. We therefore revised both the terminology and the analysis framework so that the software no longer presents a universal tonic/phasic decomposition as though the same criteria apply across sensor classes.

In the revised manuscript, phasic events are defined operationally through event detection on the corrected fluorescence signal. A separate slow-signal analysis summarizes longer-timescale fluorescence variation independently of event detection and is not interpreted as a direct measure of tonic activity.

We also expanded the biological examples to include GCaMP7s, GRAB-ACh4m, and GRAB-DA3m recordings, which differ in kinetics and baseline behavior. The revised Discussion emphasizes that the same analysis framework can be applied across sensor classes, but that correction and event-detection settings may need to be adjusted according to the characteristics of the sensor and recording.

7) Insufficient description of software functionality and workflow.

The manuscript currently provides limited explanation of the software interface, analytical options, and processing workflow. It is unclear whether the software includes integrated documentation, tutorials, or explanatory guidance for users. More comprehensive methodological detail is necessary to evaluate reproducibility and usability.

While the interface includes hover-over information prompts, these are currently too limited to guide users unfamiliar with the underlying analytical procedures. If the goal is to standardize and streamline fiber photometry analysis across users with varying levels of computational expertise, a detailed step-by-step tutorial and more comprehensive help resources are needed, particularly for preprocessing steps such as normalization, detrending, fitting, and correction parameter selection.

We agree that the original manuscript did not describe the software workflow, analytical options, or user guidance in sufficient detail. In response, we substantially revised both the software interface and the manuscript.

The revised software introduces a step-by-step guided workflow that takes the user from data selection and recording-structure definition through ROI selection, correction-strategy selection, event-detection configuration, review of the analysis plan, setup validation, execution, and inspection of completed results. Figure 1 now summarizes the workflow, and the revised Methods describe the analysis steps and outputs in substantially greater detail.

We also expanded the scientist-facing guidance that accompanies the software. The repository now includes a detailed README and a dedicated Guided Scientist Guide that explains the analytical decisions presented in the guided workflow and how users should evaluate them. The software also provides explanatory tooltips, pre-run correction and event-detection previews, defined default settings, and built-in intermittent and continuous synthetic demonstration datasets.

8) Clarification needed regarding handling of long continuous recordings Testing with a multi-day continuous recording resulted in only the first 10 minutes being displayed upon upload. This raises an important question regarding whether the software is optimized for continuous recordings spanning hours to days, as described in the manuscript, or primarily for segmented/time-lapse acquisition paradigms. If continuous recordings are supported, the authors should clarify how these are loaded, visualized, and tuned. If additional settings are required, this should be explained in the documentation. If only time-lapse data are currently supported, that limitation should be clearly stated.

We agree that the original manuscript did not adequately demonstrate or explain support for long continuous recordings. The revised software now supports continuous recordings spanning hours to days as a distinct guided workflow path.

Continuous recordings are loaded as a single uninterrupted time series, with analysis windows used for summary outputs and review. Correction is estimated across successive correction segments, while event detection is performed across the reconstructed contiguous corrected trace. The revised results interface provides continuous-recording overviews and windowed summaries so that the full recording can be reviewed.

We have also revised the Methods and user documentation to describe the continuous-recording workflow and its analysis and review options. The software now includes a built-in 48-h continuous synthetic demonstration dataset so that users can test the complete continuous workflow directly.

9) Need for comprehensive user documentation and tutorial materials A major opportunity for improvement is documentation. The app is installable and usable without Python expertise, and basic upload is straightforward, which is a strength. However, users with little programming or signal-processing experience would benefit from a much more detailed step-by-step tutorial. The current hover-over information prompts are too limited to explain the rationale behind the many available options. More extensive guidance, ideally including example datasets, walkthroughs, and troubleshooting documentation, would make the software significantly more accessible and reproducible across users.

We agree that the original documentation was not sufficient for scientists with limited programming or signal-processing experience. In response, we added a detailed README and a dedicated "Guided Scientist Guide" rather than relying primarily on hover-over prompts.

The README provides installation and launch instructions, supported-input information, a guided-workflow overview, built-in demo instructions, descriptions of saved results and limitations, and links to more detailed technical documentation. The Guided Scientist Guide provides a step-by-step scientist-facing walkthrough of the workflow and explains the analytical decisions that remain with the user. It includes detailed guidance for evaluating correction previews, selecting correction approaches, configuring and interpreting event detection, reviewing the analysis plan and completed results, and evaluating whether correction and event-detection choices are scientifically defensible.

The Guide also includes troubleshooting for common problems with data import, timing configuration, correction, event detection, execution, and results review. In addition, the software now generates built-in intermittent and continuous synthetic demonstration datasets that can be taken through the normal guided workflow without experimental data.

Minor Comments 1) Clarify file compatibility and supported acquisition systems Figure 2 suggests compatibility primarily with .rwd and .npm formats. The manuscript should explicitly state: a) which acquisition systems are supported, b) whether Neurophotometrics, Tucker Davis, Doric, and other common systems are compatible, c) whether generic .csv or .txt files can be imported, and d) clarify handling of multiple files and batch processing We have revised the manuscript and user documentation to clarify supported acquisition formats and handling of multiple recording files. The guided workflow directly supports RWD recordings, Neurophotometrics recordings, and user-mapped CSV files. RWD recordings can be analyzed as either intermittent or continuous recordings, whereas Neurophotometrics recordings are currently supported for intermittent analyses.

User-mapped CSV files can represent either multiple intermittent sessions or a single continuous recording, with the user specifying the time, signal, and reference columns for each ROI. Tucker-Davis, Doric, and other systems without dedicated importers can therefore be analyzed after export to a compatible CSV containing those required fields. Generic .txt files are not imported directly and must first be converted to the supported CSV format.

For intermittent recordings, multiple files are treated as separate sessions and retained at their corresponding positions in the experimental timeline rather than being concatenated sequentially. The software can analyze multiple ROIs within a single run. In the biological datasets presented here, these ROIs represent recordings from different animals. Multiple animals can be analyzed within a single run, with correction and event-detection settings determined independently for each ROI rather than imposed uniformly across recordings.

2) Additional detail is needed regarding: a) import of multiple sequential recording files, b) batch processing across animals, c) simultaneous visualization of multiple recordings, d) standardization of tuning across datasets, e) clarify scalability of event rejection workflows, f) the manuscript should explain whether artifact/event rejection must be performed individually for each segment or whether session-wide correction is possible for very long recordings containing many large events We have expanded the manuscript and documentation to clarify how the workflow handles multiple recordings and how correction scales across long datasets.

For intermittent recordings, multiple sequential files are treated as separate sessions and retained at their corresponding positions in the experimental timeline. Multiple ROIs, including recordings from different animals, can be included in one run, with correction and event-detection settings determined separately for each ROI. Multiple sessions from a selected ROI can be reviewed together in day-aligned and longitudinal views. Detailed inspection is performed separately for each ROI.

The guided workflow provides common default settings as a standardized starting point, while allowing correction and event-detection choices to be adjusted when individual ROIs require different treatment. The settings used for each ROI are preserved with the completed analysis.

Robust correction does not require manual rejection of individual large events. For intermittent recordings, the correction model is estimated across the full recording session. For continuous recordings, correction is estimated within successive correction segments. Robust and adaptive correction strategies automatically exclude or gate unsuitable samples during fitting within each interval, allowing long recordings containing many large excursions to be processed without manual event-by-event rejection.

3) Provide more detail regarding tuning options shown in Figure 5 The manuscript should define the tuning parameters, explain what each adjustment accomplishes, and clarify whether standardized presets or recommended settings exist.

We agree that the tuning options shown in the original Figure 5 were not described in sufficient detail. We have replaced the former post-run event-retuning workflow with pre-run event-detection configuration and preview within the guided workflow, and the revised Figure 5 now illustrates this process.

The revised Methods define the available event-detection settings, including threshold method and value, signal-excursion polarity, minimum peak distance, prominence, minimum width, and optional prefiltering. The guided workflow provides a common default profile as a standardized starting point, with settings adjusted separately for individual ROIs when needed. Users can preview detections before execution, and the settings used for each ROI are preserved with the completed analysis.

4) Clarify the "dynamic fit" procedure The functionality and rationale underlying the "dynamic fit" approach are not sufficiently explained. More methodological detail would improve interpretability and reproducibility.

We agree that the term "dynamic fit" was too vague in the original manuscript. In the revised software and manuscript, we no longer use "dynamic fit" as a general label. Instead, the correction approaches are provided as distinct strategies with explicit methodological descriptions.

The revised guided workflow includes Global Linear Regression, Robust Global Event-Reject Fit, Adaptive Event-Gated Regression, and Signal-Only F0. The Methods now describe how each strategy estimates the correction reference or baseline, the parameters that govern fitting, and how the corrected signal is calculated.

5) Clarify expected user expertise requirements It is unclear whether users are expected to understand the underlying analytical methods in detail or whether the software provides guidance sufficient for less experienced users.

We have revised the manuscript and user documentation to clarify the expected level of user expertise. Programming knowledge is not required for ordinary use of the guided workflow after installation, and the software provides a step-by-step interface, previews, default settings, explanatory guidance, and a dedicated Guided Scientist Guide to support users with limited computational or signal-processing experience. The guided workflow reduces the need for computational expertise but does not replace scientific judgment about the appropriateness of the analysis.

6) Clarify software outputs and export capabilities The manuscript should specify: a) output file formats, b) whether intermediate processing stages can be exported, c) compatibility with downstream statistical analysis workflows.

We have revised the manuscript and user documentation to clarify the software outputs and their use in downstream analysis. Figures and visualizations are saved as PNG files, while quantitative results are saved as CSVs. CSV outputs include corrected time-series data and quantitative summaries such as integrated fluorescence, event rate, and slow-signal values.

The application is intended to carry users through photometry preprocessing and quantification so that the resulting numerical outputs can be analyzed using their usual statistical workflows (GraphPad Prism, Python, MATLAB, etc.). Corrected traces and other saved analysis products can also be inspected outside the application, although the software does not expose every internal computational intermediate as a separate export.

7) The discussion would benefit from a more explicit consideration of the software's current limitations, including potential constraints related to sensor specificity, recording conditions, and user-dependent parameter optimization.

Consider expanding discussion of generalizability and limitations We agree that the original Discussion did not sufficiently address the limitations and generalizability of the workflow. We have expanded the revised Discussion to make these constraints explicit.

The revised Discussion now emphasizes that correction performance depends on the structure and stability of the signal-reference relationship and that no single correction strategy is appropriate across all recording conditions. It also clarifies that reference-based correction can reduce fluctuations shared between signal and reference, but that the origin of signal-specific excursions generally cannot be determined from fluorescence traces alone.

We likewise clarify that event-detection criteria may need to differ across sensors, circuits, and recording conditions because fluorescence transients vary in amplitude, duration, and temporal structure. Finally, although the revised manuscript demonstrates application of the workflow to calcium, acetylcholine, and dopamine sensor recordings, we emphasize that sensor kinetics and biological context affect how correction and event-detection settings should be selected and interpreted.
